# Perioperative outcomes in patients with myalgic encephalomyelitis/chronic fatigue syndrome undergoing general anesthesia: a retrospective matched-pair study

**DOI:** 10.1186/s12871-026-04102-5

**Published:** 2026-07-16

**Authors:** Felix M. Steinkirchner, Christina K. Kaufmann, Richard F. Kraus, Maximilian Käss, Elisabeth Schieffer, Bernhard M. Graf, Christoph Lassen, Viktoria Kimmerling, Alexander Dejaco

**Affiliations:** 1 Department of Anesthesiology, ME/CFS Research Group, University Hospital Regensburg, Franz-Josef-Strauß-Allee 11, Regensburg, 93053 Germany; 2 Department of Cardiology, Angiology and Intensive Care, University Hospital Gießen and Marburg, Marburg, Germany

**Keywords:** ME/CFS, Myalgic Encephalomyelitis, Chronic Fatigue Syndrome, General Anesthesia, Intraoperative Hypotension, Postoperative Pain, Dysautonomia, Perioperative outcomes, Autonomic dysfunction, Matched-pair analysis

## Abstract

**Background:**

Myalgic encephalomyelitis/chronic fatigue syndrome (ME/CFS) is a chronic multisystem disease characterized by profound fatigue, post-exertional malaise, cognitive impairment, and autonomic dysfunction. Despite features with potential relevance for anesthesia and perioperative care, empirical data on perioperative outcomes in patients with ME/CFS remains limited. We therefore performed a retrospective matched-pair analysis to generate clinical data on perioperative responses and identify areas for future research.

**Methods:**

We conducted a retrospective matched-pair analysis at a single tertiary center. All patients with ME/CFS undergoing general anesthesia from 2015 to 2026 were identified using ICD-10-GM codes with additional manual verification and matched 1:1 to controls for comparison. Patients with confounding diagnoses or American Society of Anesthesiologists physical status above III were excluded. The analysis focused on intraoperative hemodynamic parameters, including baseline, post-induction, median, and lowest recorded systolic blood pressure and heart rate, as well as early postoperative outcomes in the post-anesthesia care unit (PACU), including maximum pain scores and requirement for rescue analgesia.

**Results:**

Out of 189 individuals identified through ICD-10 codes, 15 matched pairs were included after application of exclusion criteria. Patients with ME/CFS exhibited lower minimum intraoperative systolic blood pressure (90.0 [82.5–95.0] vs. 100.0 [90.0–110.0] mmHg, *p* = 0.044) and lower minimum heart rate (50.0 [40.0–57.5] vs. 60.0 [50.0–65.0] bpm, *p* = 0.012). Vasopressor use and fluid administration did not differ, and no episodes of severe hypotension or perioperative adverse events were observed. Postoperative pain was higher in ME/CFS, with higher maximum pain scores (NRS 5.0 [4.0–6.0] vs. 1.0 [0.0–4.0], *p* = 0.008) and more frequent opioid rescue analgesia (80% vs. 33%, *p* = 0.039). Postoperative nausea or vomiting, oxygen supplementation, and PACU length of stay were similar between groups.

**Conclusions:**

In this small exploratory cohort, general anesthesia was not associated with clinically relevant hemodynamic instability in patients with ME/CFS. Postoperative pain scores and opioid rescue requirements were higher in the ME/CFS group. Post-exertional malaise, a key disease feature with potentially delayed onset and significant impact, was not captured and remains an important target for future research. These findings should be considered hypothesis-generating and support prospective studies evaluating perioperative management and patient-relevant outcomes in ME/CFS.

**Supplementary Information:**

The online version contains supplementary material available at 10.1186/s12871-026-04102-5.

## Introduction

Myalgic encephalomyelitis/chronic fatigue syndrome (ME/CFS) is a chronic multisystem disease characterized by post-exertional malaise (PEM), debilitating fatigue, cognitive dysfunction, and dysautonomia [1], affecting an estimated 0.68% of the population [2]. The pathophysiology remains incompletely understood and likely involves complex interactions between autonomic, immune, and metabolic dysfunction [3–5].

Several features of ME/CFS have direct relevance for perioperative care. Autonomic dysfunction with altered cardiovascular regulation is common and frequently manifests as orthostatic intolerance (OI) or postural orthostatic tachycardia syndrome (POTS), characterized by inappropriate vascular tone and exaggerated tachycardic responses [6]. A relevant subset of patients experiences OI or POTS, which may lead to suboptimal hemodynamic responses under anesthesia [7, 8]. Anesthetic agents inherently induce vasodilation and myocardial depression, effects that may be amplified by pre-existing autonomic dysregulation [9, 10]. This may predispose to hypotension, impaired tolerance to hypovolemia, or abnormal cardiovascular responses to surgical positioning and conditions that reduce venous return, such as ventilation with positive end-expiratory pressure, steep anti-Trendelenburg or pneumoperitoneum during laparoscopic procedures [11, 12]. Neuropathy and heightened nociceptive processing, including mechanisms such as central sensitization, may contribute to increased perioperative pain perception and greater analgesic requirements, with a subsequent higher risk of related adverse effects [13–15]. Additional features reported in subsets of patients - including gastrointestinal dysmotility, immune and mast-cell dysregulation, and neuromuscular fatigue - could theoretically influence perioperative medication handling, hypersensitivity responses, airway reflexes, or recovery from anesthesia [16–20]. Finally, PEM - a defining feature of ME/CFS - raises the possibility of impaired postoperative recovery and prolonged functional decline after surgical stress [21, 22].

Available evidence of the perioperative impact of ME/CFS is limited and consists largely of small case series and expert opinion [21, 23–27]. In the only published clinical case series to date, Fisher and Rose reported perioperative outcomes in 27 individuals with a history or perceived risk of adverse reactions after anesthesia in the context of chronic fatigue syndrome or idiopathic environmental intolerance, describing postoperative symptom exacerbations but no major intraoperative complications [27]. However, this cohort is unlikely to adequately represent the baseline ME/CFS population and did not include a control group.

Consequently, robust clinical evidence to guide perioperative management or anesthetic techniques in patients with ME/CFS remains lacking. To date, no study has systematically examined intraoperative hemodynamic stability or postoperative pain in a matched cohort of patients with ME/CFS.

Despite this, ME/CFS remains markedly under-represented in the anesthesia literature, and perioperative care is largely delivered as “care as usual” rather than being informed by disease-specific evidence. This represents a substantial unmet clinical need, as the absence of robust data limits the ability to identify perioperative factors that may exacerbate symptoms and hinders the development of tailored anesthetic strategies. Generating clinical data in this area is therefore essential to characterize perioperative responses in ME/CFS and to inform future prospective studies evaluating modifiable anesthesia-related factors, including choice of anesthetic technique.

We therefore conducted a retrospective matched-pair analysis to characterize perioperative responses, including intraoperative hemodynamic parameters and postoperative pain, in patients with ME/CFS compared with matched controls undergoing general anesthesia.

## Methods

### Study design and patient identification

We conducted a retrospective single-center analysis at the University Hospital Regensburg covering the period from January 2015 to January 2026. All patients with a documented diagnosis of ME/CFS (International Classification of Diseases 10th Revision, German Modification (ICD-10-GM) code G93.3) or post-COVID syndrome (ICD-10-GM code U09.9) and a recorded surgical procedure were identified through the institutional clinical information system (CIS).

For patients coded with G93.3, the diagnosis was accepted as established without further verification. For patients coded with U09.9, medical records, including discharge letters, were manually reviewed, as this code encompasses a heterogeneous post-COVID population in which ME/CFS is not uniformly present. This step was necessary because a proportion of patients with ME/CFS in the institutional CIS were coded exclusively as U09.9 without an accompanying G93.3-code, while the majority of U09.9-coded patients did not fulfill criteria for ME/CFS. Consequently, all included cases in the subsequent analyses had a physician-documented diagnosis of ME/CFS at the time of anesthesia, either formally coded as G93.3 or identified through manual review of medical records in patients coded exclusively as U09.9. Formal diagnostic criteria such as the Canadian Consensus Criteria [22] were not applied.

### Exclusion criteria

Cases were excluded if they had not undergone general anesthesia for their procedure, if confounding diagnoses were present (malignant, neurodegenerative, or decompensated illness, operationalized as an American Society of Anesthesiologists (ASA) physical status score > III), or if the ME/CFS diagnosis had only been established after the surgical procedure. If multiple procedures were recorded for a patient, only the most recent procedure was included, as ME/CFS diagnosis could not in every case be assumed to have been present at the time of earlier procedures.

### Matching

Each eligible case with ME/CFS was matched 1:1 to a control subject without ME/CFS using two formal criteria: sex and surgical procedure (same or clinically comparable). Surgical procedure was selected as a formal matching criterion because procedure type was considered the most relevant available determinant of perioperative physiology, surgical stress, patient positioning, expected postoperative pain, and anesthetic requirements. When an identical procedure was not available, the most clinically comparable available procedure was selected pragmatically by the study team based on overall clinical similarity, considering these characteristics. Additional variables were therefore not used as formal matching criteria to avoid over-restriction of eligible pairs. Instead, relevant baseline and procedure-related characteristics, including age, ASA physical score, body mass index (BMI), anesthetic technique, and year of surgery, were assessed post hoc to evaluate comparability between groups.

### Data extraction

Data were extracted from hand-written anesthetic, post-anesthesia care unit (PACU) records and discharge letters. Intraoperative vital signs were routinely recorded at five-minute intervals. Extracted parameters included intraoperative fluid use, vasopressor use (maximum norepinephrine infusion rate during continuous infusion), total administered anesthetic and opioid dose and averaged opioid dose per time, duration of anesthesia, time from end of surgery to extubation, and hemodynamic trajectories.

Pre-admission medication was extracted from available records.

### Endpoints

In keeping with the exploratory nature of this study, no single primary endpoint was designated; instead, two pre-specified domains were defined a priori as the focus of the analysis: intraoperative hemodynamic stability, assessed through post-induction, median intraoperative, nadir, and lowest sustained values for both systolic blood pressure and heart rate, as well as postoperative pain intensity, defined as the highest numerical rating scale (NRS) pain score recorded in the PACU. Baseline blood pressure and heart rate were defined as the last recorded values prior to anesthetic induction. The intraoperative nadir was defined as the single lowest value recorded at any time point; the lowest sustained value as the lowest value confirmed across consecutive measurements spanning at least 15 min.

Secondary outcomes included vasopressor requirements, intraoperative opioid consumption, time to extubation, PACU stay duration, opioid rescue analgesia, defined as any opioid administration in the PACU (piritramide, oxycodone, or hydromorphone), non-opioid rescue analgesia (metamizole, ibuprofen, parecoxib, paracetamol), postoperative nausea and vomiting (PONV), supplemental oxygen requirement, vital signs in the PACU at admission and discharge, and postoperative hospital length of stay.

### Statistical analysis

Statistical analysis was performed in R (version 4.5.1; R Foundation for Statistical Computing, Vienna, Austria). Matched pairs were compared using the Wilcoxon signed-rank test for continuous variables and McNemar’s test for binary outcomes. Given the small sample size and the matched-pair design, no multivariable regression modeling was performed in order to avoid overfitting; analyses were therefore restricted to matched-pair comparisons. A p-value < 0.05 was considered statistically significant. Given the exploratory design and the number of comparisons performed, statistically significant findings should be regarded as hypothesis-generating, and the possibility that some represent chance findings due to multiple testing cannot be excluded. No formal sample size calculation was performed because of the exploratory design; instead the study included all eligible patients identified during the study period. This approach was chosen because the study aimed to describe the complete available experience at a single center over an eleven-year period and to generate hypotheses for future prospective studies, and no prior data existed on which to base a meaningful power calculation. Variables with missing values are indicated in the respective tables; no imputation was performed. Continuous variables were analyzed on their original scale without categorization. No subgroup analyses, interaction tests, or formal outlier exclusion were performed given the small sample size; individual data points are displayed in all figures. No sensitivity analyses were performed.

### Potential sources of bias

Several potential sources of bias inherent to this study design should be considered. First, case identification relied on physician-documented diagnoses and ICD-10 coding rather than a standardized case definition applied prospectively. Although only patients with a documented clinical diagnosis of ME/CFS at the time of anesthesia were included, the specific diagnostic criteria applied in routine care could not be verified retrospectively; diagnostic heterogeneity or misclassification can therefore not be excluded. Second, severity selection bias is inherent to a surgical cohort: patients with the most severe ME/CFS are less likely to undergo elective procedures and are therefore systematically under-represented. Third, the five-minute interval manual hemodynamic documentation introduces measurement imprecision, particularly for transient hemodynamic events.

## Results

### Study population and perioperative characteristics

**Fig. 1:**
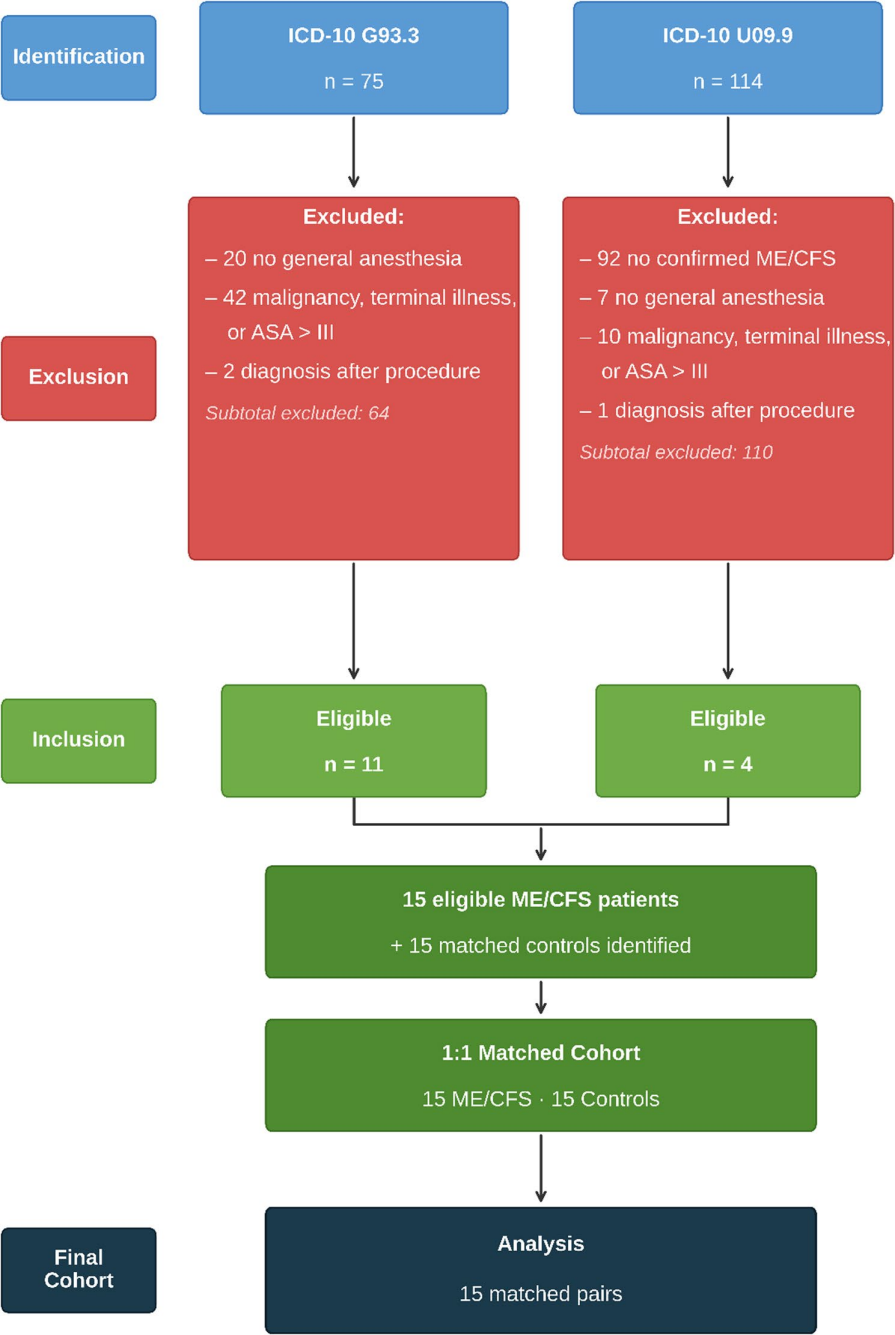
Flow diagram of participant identification and selection. ICD-10 = International Classification of Diseases, 10th Revision, German Modification; ASA = American Society of Anesthesiologists; ME/CFS = myalgic encephalomyelitis/chronic fatigue syndrome. Controls were matched 1:1 by sex and surgical procedure type. Where a patient had undergone multiple procedures, only the most recent was included. Patients may fulfill more than one exclusion criterion; only the first applicable criterion was counted

Extraction of cases based on ICD-10 codes yielded 75 and 114 patients who underwent a surgical procedure with a diagnosis of G93.3 or U09.9, respectively. From those with a G93.3 diagnosis, 20 were excluded because no general anesthesia was performed and 42 were excluded due to malignancy, terminal illness, or ASA status > III. Of the remaining 13 cases, 2 were excluded because the diagnosis of ME/CFS had only been established after the surgical procedure and was therefore not present at the time of anesthesia, leaving 11 eligible cases in the G93.3 cohort. Out of patients with a U09.9 diagnosis, 92 were excluded as ME/CFS was not confirmed in their medical records, 7 because no general anesthesia was performed, 10 due to malignancy, terminal illness, or ASA status above III, and 1 because the ME/CFS diagnosis had only been established after the surgical procedure. This left 4 eligible patients in the U09.9 cohort. This resulted in 15 eligible ME/CFS cases overall, which were matched with 15 controls (Fig. 1). All subsequent analyses refer to this cohort.

No eligible cases were found between 2019 and 2021, coinciding with the COVID-19 pandemic and the associated reduction in elective surgical activity. Case numbers increased markedly from 2022 onwards, paralleling the rising clinical awareness of ME/CFS in the context of post-COVID.

Baseline characteristics were comparable between groups (Table 1). No significant differences were observed for any baseline characteristic (all *p* ≥ 0.196), except for the year of the procedure (*p* = 0.003). The cases in the ME/CFS cohort were unevenly distributed across the study period, however, matched cohorts were well aligned (see Additional file 1). Out of 15 pairs, 11 underwent surgery within one year of each other with an overall median offset of 8.3 months and an IQR of 3.1–15.8. Residual confounding from evolving perioperative practice can nonetheless not be fully excluded. Surgical procedures were heterogeneous overall, but also well aligned between matched cohorts (Additional file 2). Regular home medication is summarized in Additional file 3, with data available for 28/30 patients. Given the small sample size, these data were interpreted descriptively.

**Table 1:** Baseline characteristics

Variable	ME/CFS (*n* = 15)	Control (*n* = 15)	*p*-value
Demographics			
Age (years)	47.7 (41.8–60.3)	49.5 (37.8–61.7)	0.629
Female sex, n (%)	11/15 (73.3%)	11/15 (73.3%)	—
BMI (kg/m²)	29.7 (23.9–34.5)	26.9 (25.0–31.0)	0.887
Weight (kg)	86.0 (67.0–105.0)	80.0 (68.5–90.5)	0.267
Height (cm)	174.0 (165.0–178.0)	168.0 (161.5–175.0)	0.196
*Clinical status*			
ASA status (I/II/III)	0/8/7	1/9/5	—
History of PONV, n (%)	4/15 (26.7%)	2/15 (13.3%)	0.687
Preoperative anxiolysis, n (%)	2/15 (13.3%)	3/15 (20.0%)	1.000
*Procedure*			
Additional regional anesthesia, n (%)	1/15 (6.67%)	1/15 (6.67%)	—
Anesthesia duration (min)	115.0 (95.0–130.0)	100.0 (67.5–160.0)	0.459
Year of procedure	2024 (2020–2024)	2025 (2022–2025)	0.003 *
*Anesthesia type*			
Inhaled anesthetics, n (%)	12/15 (80.0%)	13/15 (86.7%)	—
TIVA, n (%)	3/15 (20.0%)	2/15 (13.3%)	—

Baseline characteristics of the investigated cohort with myalgic encephalomyelitis/chronic fatigue syndrome (ME/CFS) as compared to the matched control cohort

*BMI* body mass index, *PONV* postoperative nausea and vomiting, *ASA* American Society of Anesthesiologists, *TIVA* total intravenous anesthesia

Continuous variables are presented as median and interquartile range (IQR); binary variables as n/N (%); — = not tested (matching variable or descriptive comparison only)

* *p*<0.05

No previous anesthesia-related complications were documented in any of the included patients. A history of postoperative nausea and vomiting was present in 4 of 15 patients with ME/CFS (26.7%) and 2/15 controls (13.3%). Preoperative regional anesthesia was performed in one patient in each group. Anesthesia and surgical duration, as well as time from end of surgery to extubation, were similar between groups. Postoperative hospital length also did not differ between groups.

### Anesthetic management and intraoperative hemodynamics

Parameters of anesthetic management did not differ between groups (Table 2). Sufentanil was administered in 5 patients with ME/CFS and 6 controls; Remifentanil was used in 10 patients with ME/CFS and 9 controls. Two cases per group were excluded from remifentanil infusion rate comparisons (*n* = 1 per group administered via target-controlled infusion, *n* = 1 per group documented in µg·kg⁻¹·min⁻¹), leaving 13 analyzable pairs. Piritramide was used in 3 patients with ME/CFS and no controls, while oxycodone was given in 6 patients with ME/CFS (40.0%) and 8 controls (53.3%). Neither intraoperative piritramide nor oxycodone use differed significantly between groups. End-tidal volatile anesthetic concentrations (in minimum alveolar concentration equivalents) were similar between groups. Intraoperative fluid administration and vasopressor management, including the proportion of patients receiving vasopressors and the maximum norepinephrine infusion rate, were comparable between groups.

**Table 2:** Anesthetic management and intraoperative hemodynamics

Variable	ME/CFS (*n* = 15)	Control (*n* = 15)	*p*-value
Anesthetic management			
Propofol induction dose (mg)	200.0 (185.0–200.0)	200.0 (165.0–265.0)	0.610
Propofol maintenance rate (mg/h)	570.0 (555.0–585.0)	620.0 (590.0–650.0)	—
Sufentanil use, n (%)	5/15 (33.3%)	6/15 (40.0%)	1.000
Sufentanil total (µg)	0.0 (0.0–38.8)	0.0 (0.0–35.0)	0.293
Sufentanil per hour (µg/h)	0.0 (0.0–14.4)	0.0 (0.0–19.2)	0.093
Piritramide use, n (%)	3/15 (20.0%)	0/15 (0.0%)	0.250
Piritramide total dose (mg)	0.0 (0.0–0.0)	0.0 (0.0–0.0)	0.181
Oxycodone use, n (%)	6/15 (40.0%)	8/15 (53.3%)	0.500
Oxycodone total dose (mg)	0.0 (0.0–5.0)	4.0 (0.0–5.0)	0.785
Remifentanil use, n (%)	10/15 (66.7%)	9/15 (60.0%)	1.000
Remifentanil infusion rate (mg/h)	1.20 (0.00–1.20)	1.00 (0.00–1.20)	0.361
End-tidal MAC equivalent	0.8 (0.8–0.8)	0.7 (0.7–0.8)	0.203
Total intraoperative fluid (×500 ml)	2.0 (1.5–3.0)	2.0 (1.0–2.0)	0.227
Any vasopressor use, n (%)	12/15 (80.0%)	13/15 (86.7%)	1.000
Norepinephrine maximum (mg/h)	0.2 (0.1–0.5)	0.3 (0.2–0.4)	0.932
Time end surgery → extubation (min)	5.0 (0.0–5.0)	5.0 (5.0–7.5)	0.340
*Intraoperative blood pressure*			
Baseline systolic BP (mmHg)	140.0 (130.0–150.0)	140.0 (130.0–155.0)	0.336
Post-induction systolic BP (mmHg)	110.0 (95.0–137.5)	110.0 (100.0–140.0)	0.439
Median systolic BP (mmHg)	110.0 (110.0–120.0)	110.0 (110.0–120.0)	0.720
Absolute systolic BP nadir (mmHg)	90.0 (82.5–95.0)	100.0 (90.0–110.0)	0.044 *
Lowest sustained systolic BP > 15 min (mmHg)	100.0 (90.0–110.0)	110.0 (100.0–110.0)	0.301
Sustained hypotension (sys < 80 mmHg, > 15 min), n (%)	1/15 (6.67%)	0/15 (0.0%)	1.000
Severe hypotension (sys < 65 mmHg, any), n (%)	0/15 (0.0%)	0/15 (0.0%)	—
*Intraoperative heart rate*			
Baseline HR (bpm)	75.0 (70.0–80.0)	80.0 (80.0–82.5)	0.134
Post-induction HR (bpm)	65.0 (60.0–67.5)	70.0 (60.0–80.0)	0.180
Median HR (bpm)	60.0 (50.0–70.0)	70.0 (60.0–75.0)	0.053
HR nadir (bpm)	50.0 (40.0–57.5)	60.0 (50.0–65.0)	0.012 *
Lowest sustained HR > 15 min (bpm)	50.0 (47.5–60.0)	60.0 (52.5–70.0)	0.024 *
Sustained bradycardia (HR < 45 bpm, > 15 min), n (%)	3/15 (20.0%)	1/15 (6.67%)	0.625

Anesthetic management and intraoperative hemodynamic parameters of patients with ME/CFS and matched controls (n=15 matched pairs). Continuous variables are presented as median (IQR); binary variables as n/N (%). Missing values: end-tidal MAC (controls n=2, ME/CFS n=3), sufentanil (controls n=2, ME/CFS n=3), propofol induction dose (ME/CFS n=1). Propofol maintenance rate reported for patients receiving continuous propofol infusion only (ME/CFS n=2, controls n=2). Remifentanil infusion rate: TCI cases (n=1 per group) and cases documented in µg·kg⁻¹·min⁻¹ (n=1 per group) excluded from rate calculation. — = not tested

*MAC* minimum alveolar concentration, *bpm* beats per minute, *BP* blood pressure, *HR* heart rate

* *p*<0.05

**Fig. 2:**
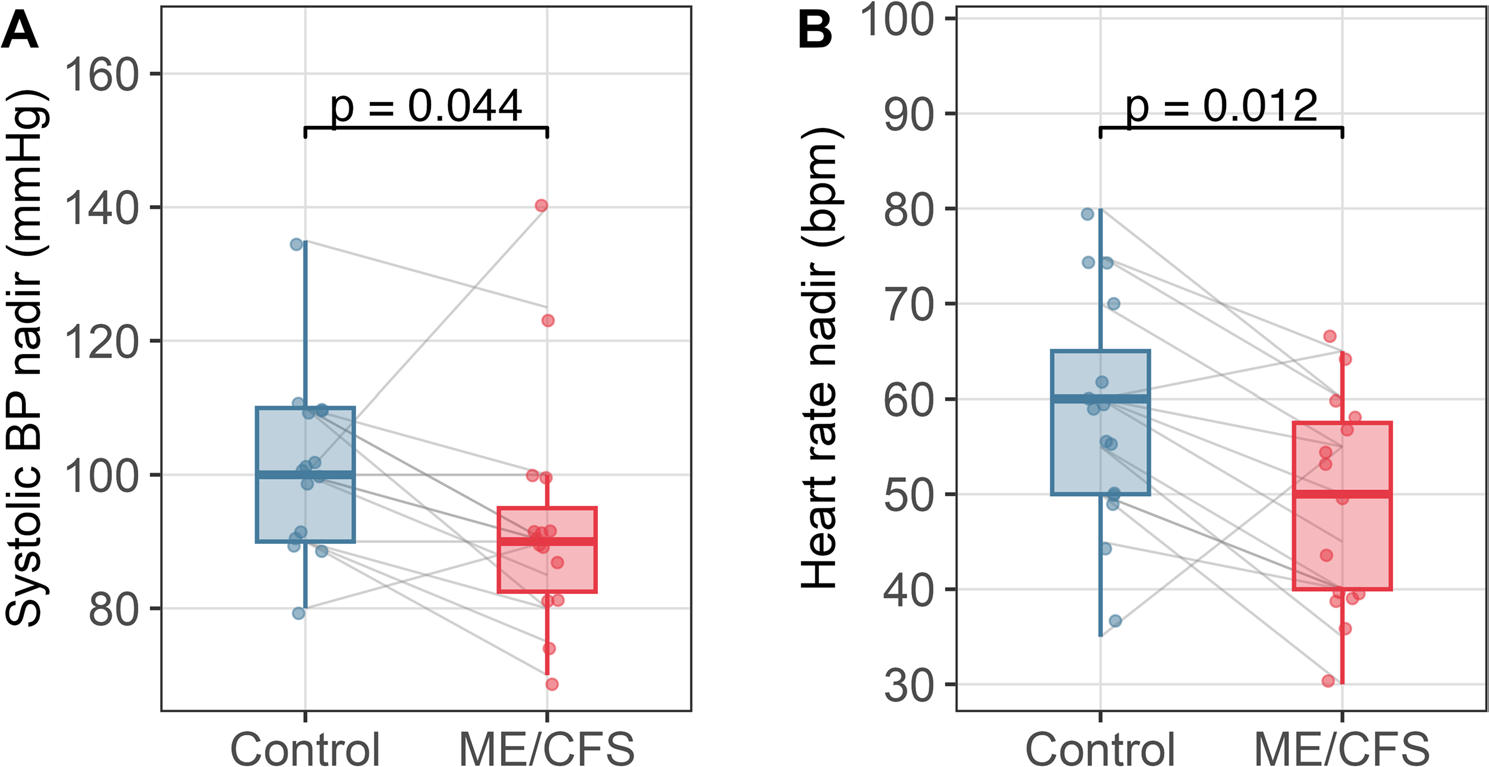
Boxplots of lowest recorded intraoperative systolic blood pressure (**A**) and heart rate (**B**) in patients with myalgic encephalomyelitis/chronic fatigue syndrome (ME/CFS) and matched controls undergoing general anesthesia (*n* = 15 matched pairs). Lines connect matched pairs

Intraoperative hemodynamic parameters were largely similar between groups (Table 2). Pre-induction blood pressure, post-induction blood pressure, and median intraoperative blood pressure did not differ significantly. However, the lowest recorded systolic blood pressure was significantly lower in patients with ME/CFS compared with controls (median 90.0 [IQR 82.5–95.0] vs. 100.0 [IQR 90.0-110.0] mmHg; *p* = 0.044; Fig. 2-A), as was the lowest recorded heart rate (median 50.0 [IQR 40.0-57.5] vs. 60.0 [IQR 50.0–65.0] bpm; *p* = 0.012; Fig. 2-B). Importantly, no episodes of severe hypotension (systolic blood pressure < 65 mmHg) occurred in either group.

### Post-anesthesia care unit outcomes

**Fig. 3:**
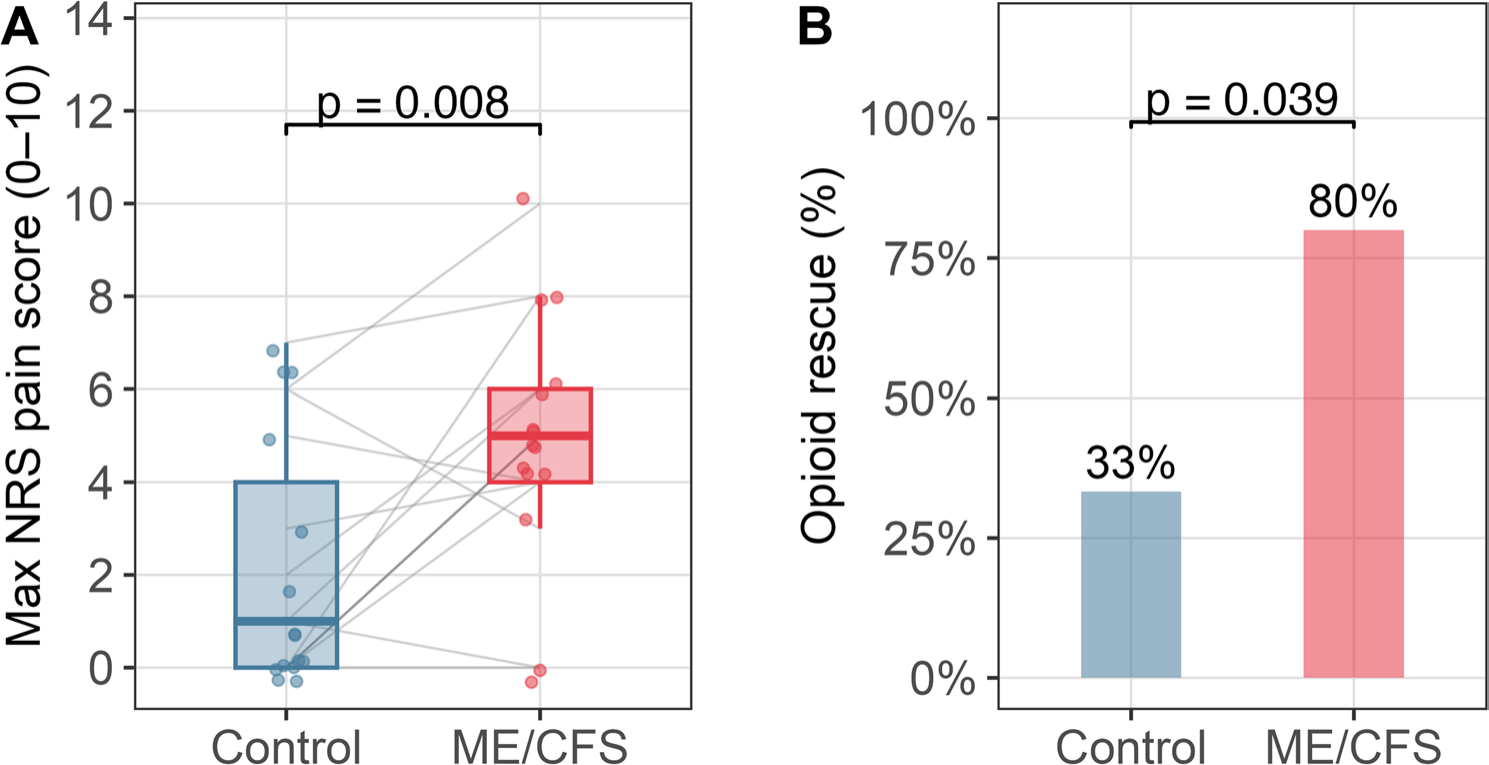
Post-anesthesia care unit pain ratings in patients with myalgic encephalomyelitis/chronic fatigue syndrome (ME/CFS) and matched controls after general anesthesia (*n* = 15 matched pairs). (**A**) Maximum numerical rating scale (NRS) pain score recorded during post-anesthesia care (0 = no pain, 10 = worst imaginable pain). (**B**) Proportion of patients requiring opioid rescue analgesia. Lines connect matched pairs

In the PACU, patients with ME/CFS reported significantly higher maximum pain scores compared with controls (Fig. 3-A) (Table 3).

**Table 3:** Post-anesthesia care unit outcomes

Variable	ME/CFS (*n* = 15)	Control (*n* = 15)	*p*-value
Pain			
Maximum NRS pain score (0–10)	5.0 (4.0–6.0)	1.0 (0.0–4.0)	0.008 *
*Analgesia*			
Opioid rescue analgesia, n (%)	12/15 (80.0%)	5/15 (33.3%)	0.039 *
Non-opioid rescue analgesia, n (%)	6/15 (40.0%)	5/15 (33.3%)	1.000
*Other complications*			
PONV event, n (%)	1/15 (6.67%)	2/15 (13.3%)	1.000
Oxygen supplementation at discharge, n (%)	7/15 (46.7%)	5/15 (33.3%)	0.625
*Recovery*			
PACU stay duration (min)	60.0 (52.5–95.0)	70.0 (57.5–85.0)	0.861
*Vital signs in the PACU*			
Systolic BP at admission (mmHg)	130.0 (120.0–160.0)	130.0 (120.0–147.5)	0.286
Systolic BP at discharge (mmHg)	130.0 (120.0–140.0)	130.0 (120.0–140.0)	1.000
HR at admission (bpm)	90.0 (81.2–90.0)	80.0 (70.0–90.0)	0.203
HR at discharge (bpm)	80.0 (80.0–82.5)	70.0 (60.0–86.2)	0.078

Post-anesthesia care unit (PACU) outcomes in patients with myalgic encephalomyelitis/chronic fatigue syndrome (ME/CFS) and matched controls (n=15 matched pairs). Continuous variables are presented as median (IQR); binary variables as n/N (%). Missing values: PACU vital signs at admission (controls n=1), heart rate at discharge (controls n=3, ME/CFS n=3)

*BP* blood pressure, *bpm* beats per minute, *HR* heart rate, *IQR* interquartile range, *mmHg* millimeters of mercury, *NRS* numerical rating scale, *PACU* post-anesthesia care unit, *PONV* postoperative nausea and vomiting

* *p*<0.05

Opioid rescue analgesia was required in 12 of 15 patients with ME/CFS (80.0%) compared with 5 of 15 control patients (33.3%), representing a statistically significant difference (*p* = 0.039), (Fig. 3-B). The absolute risk difference for opioid rescue analgesia was 46.7%. Preoperative opioid use occurred exclusively in the ME/CFS group (4/13 vs. 0/15). Psychotropic medication use was also more frequent in the ME/CFS group (8/13 vs. 2/15).

In one patient with ME/CFS undergoing lower-limb debridement, postoperative pain remained poorly controlled despite systemic analgesia and could only be adequately controlled after placement of a distal sciatic nerve catheter in the PACU.

Other PACU outcomes, including non-opioid rescue analgesia, oxygen supplementation, postoperative nausea and vomiting, and PACU length of stay, did not differ significantly between groups.

## Discussion

This retrospective matched-pair analysis provides clinical data on perioperative outcomes in patients with ME/CFS undergoing general anesthesia. ME/CFS represents a potentially vulnerable population that remains underrepresented in the anesthesia literature and for whom evidence-based perioperative guidance is currently lacking. Overall, no clinically relevant hemodynamic instability was observed, whereas postoperative recovery, particularly pain, appeared more affected compared with matched controls.

Intraoperatively, patients with ME/CFS exhibited lower lowest recorded systolic blood pressure and heart rate values compared with matched controls. However, the magnitude of these differences was small, and the findings did not translate into clinically relevant hemodynamic instability. No severe adverse events or escalation of therapy were observed. Vasopressor use, fluid administration, and other hemodynamic parameters were comparable between groups, and no episodes of clinically significant hypotension occurred. Intraoperative care followed institutional standards based on guideline-conform anesthetic and hemodynamic monitoring. Continuous low-dose norepinephrine infusion is routinely used during general anesthesia at our institution, including in low-risk patients, which may have contributed to the overall hemodynamic stability observed. Thus, the hemodynamic differences should be interpreted as exploratory physiological observations rather than clinically actionable findings. Given the small sample size, this study cannot reliably assess the incidence of rare perioperative adverse events.

Postoperative recovery appeared more affected. Patients with ME/CFS reported higher pain scores and required opioid rescue analgesia more frequently in the PACU. In one patient with ME/CFS, postoperative analgesic therapy had to be escalated with placement of a distal sciatic nerve catheter due to insufficient pain control with systemic medication alone. These findings suggest a clinically relevant signal regarding early postoperative pain and may have implications for future studies of perioperative analgesic strategies in this population. Potential contributors include altered nociceptive processing, central sensitization, which have been described in ME/CFS and related conditions [14, 15], as well as sensory hypersensitivity, higher baseline pain burden and chronic analgesic medication use. Surgical tissue injury, perioperative stress, inflammation, immobilization, and sleep disruption could also plausibly interact with pre-existing pain sensitization and contribute to increased postoperative pain perception. However, because standardized preoperative assessments of pain, anxiety, pain catastrophizing, and sensory sensitivity were not available, the underlying mechanisms cannot be distinguished in this retrospective study.

Overall, evidence on anesthesia in ME/CFS remains extremely limited. Our findings are broadly consistent with the existing literature [27], suggesting that clinically relevant intraoperative hemodynamic instability may not be common in patients with ME/CFS, although robust data remain lacking. Importantly, patient-relevant outcomes such as post-exertional malaise and symptom exacerbation remain largely unstudied and require dedicated investigation. The potential impact of anesthetic techniques and perioperative management strategies in ME/CFS remains largely unexplored. Data on alternatives to general anesthesia, such as regional or neuraxial techniques alone or in combination with sedation or general anesthesia, are lacking. It is conceivable that the choice of anesthetic technique, pharmacological regimen, and perioperative care strategies may influence not only immediate postoperative outcomes but also medium- and longer-term symptom trajectories, including the risk of symptom exacerbation.

Crucially, post-exertional malaise - the hallmark feature of ME/CFS [28] - typically manifests with delayed onset and is not captured by routine perioperative documentation focused on immediate outcomes. This has important implications not only for the assessment of anesthetic techniques and perioperative management strategies, but also for clinical practice, particularly in the context of repeated or staged interventions, where patients may undergo subsequent procedures while already in a state of post-exertional malaise. Whether early postoperative pain and analgesic requirements are associated with subsequent post-exertional symptom exacerbation cannot be determined from the present data.

Prospective studies incorporating longitudinal follow-up are therefore essential to adequately capture this dimension of perioperative risk.

This study has several limitations. The retrospective single-center design, reliance on handwritten anesthetic records with five-minute hemodynamic documentation, and small sample size of 15 matched pairs limit statistical power and constrain the conclusions that can be drawn, particularly regarding rare adverse events. Case identification based on ICD-10 codes without systematic verification against established diagnostic criteria such as the Canadian Consensus Criteria [22] introduces potential misclassification. The number of identified cases was also substantially lower than expected based on population prevalence estimates, likely reflecting both underdiagnosis of ME/CFS and limitations of coding-based case identification, as well as more severely affected patients avoiding elective procedures out of concern for post-exertional malaise; the true perioperative burden of ME/CFS is therefore likely underestimated. However, the objective of this study was not to provide precise incidence estimates or fully characterize the perioperative ME/CFS population, but to identify perioperative patterns and signals warranting further investigation; these limitations primarily affect generalizability, while the internal matched comparison remains informative.

The matching strategy represents a further limitation: procedures were identical or nearly identical in 14 of 15 matched pairs, with one pair differing in procedure type (dental extraction vs. mandibular cystectomy). Residual confounding due to this procedural heterogeneity or surgeon-specific factors cannot be excluded. Similarly, unmatched disease-specific factors - including comorbidity burden, disease severity, autonomic dysfunction, and comorbid chronic pain syndromes or medication use - could not be accounted for. The group difference in year of procedure is likely exaggerated by calendar-year discretization rather than a meaningful temporal gap, but residual confounding from evolving perioperative practice cannot be fully excluded.

Baseline assessments of pain intensity, pain catastrophizing, depression, anxiety, and pain sensitivity were unavailable, precluding adjustment for baseline differences. Preoperative chronic medication was also more common in the ME/CFS group (Additional file 3), further limiting interpretation of the observed pain differences given the sample size. It therefore remains uncertain whether the observed pain differences reflect altered perioperative nociception or pre-existing between-group differences. Post-exertional malaise - arguably the most clinically relevant outcome in ME/CFS - could not be assessed in this retrospective design.

The study was not powered to detect small-to-moderate effect sizes, and non-significant findings should not be interpreted as evidence of equivalence; the findings should therefore be interpreted as hypothesis-generating signals only, requiring confirmation in larger prospective cohorts. Findings from this single-center cohort may also not be generalizable to other settings or to ME/CFS populations with different severity profiles or comorbidity patterns.

Despite these limitations, this exploratory matched analysis provides clinically relevant signals in a field with very limited existing evidence and identifies key areas for future prospective studies aimed at improving perioperative management in patients with ME/CFS.

## Conclusion

This retrospective matched analysis provides clinical data on perioperative responses in patients with ME/CFS, a population for whom evidence in anesthesia is extremely limited. General anesthesia was not associated with clinically relevant hemodynamic instability in this small cohort. Although statistically significant differences in lowest recorded blood pressure and heart rate were observed, no corresponding increase in treatment requirements or adverse events was identified. In contrast, postoperative pain scores and opioid requirements were higher, suggesting a potential vulnerability requiring careful analgesic management. Future studies should address the influence of anesthetic techniques and evaluate post-exertional malaise as a key disease feature, including its relevance for perioperative management and delayed outcomes. These hypothesis-generating findings support the need for prospective studies to better characterize perioperative risk and evaluate strategies to optimize anesthetic care in this vulnerable population.

## Supplementary Information

Additional file 1. Year of procedure for ME/CFS patients and matched controls. Boxplot showing the distribution of procedure years in the 15 matched pairs.

Additional file 2. Characteristics of matched pairs. Baseline and procedural characteristics of all 15 matched pairs.

Additional file 3. Preoperative medication classes. Summary of regular preoperative medication classes in ME/CFS patients and matched controls.

## Data Availability

The data that support the findings of this study are not publicly available due to dataprotection and privacy regulations but are available from the corresponding authorupon reasonable request, subject to applicable data protection regulations.
