# Supplementary material for: Perioperative outcomes in patients with myalgic encephalomyelitis/chronic fatigue syndrome undergoing general anesthesia: a retrospective matched-pair study
Source: BMC Anesthesiol. 2026 Jul 16;26:426. doi: 10.1186/s12871-026-04102-5 (PMC13374260; doi:10.1186/s12871-026-04102-5)
Supplement: Supplementary file 1 — Additional file 1. Year of procedure for ME/CFS patients and matched controls. Boxplot showing the distribution of procedure years in the 15 matched pairs. [file 12871_2026_4102_MOESM1_ESM.docx]

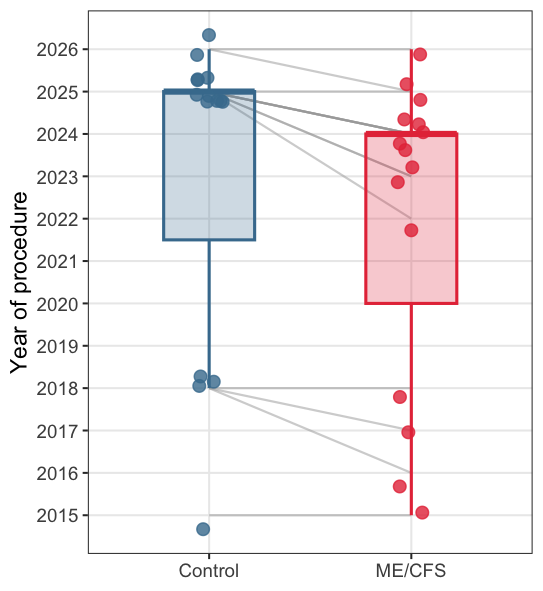


**Additional file 1.** Year of procedure for patients with myalgic encephalomyelitis/chronic fatigue syndrome (ME/CFS) and matched controls (n=15 matched pairs). Each box represents the median and interquartile range; individual data points are shown as dots; lines connect matched pairs. ME/CFS: myalgic encephalomyelitis/chronic fatigue syndrome.
