# Supplementary material for: Perioperative outcomes in patients with myalgic encephalomyelitis/chronic fatigue syndrome undergoing general anesthesia: a retrospective matched-pair study
Source: BMC Anesthesiol. 2026 Jul 16;26:426. doi: 10.1186/s12871-026-04102-5 (PMC13374260; doi:10.1186/s12871-026-04102-5)
Supplement: Supplementary file 2 — Additional file 2. Characteristics of matched pairs. Baseline and procedural characteristics of all 15 matched pairs. [file 12871_2026_4102_MOESM2_ESM.docx]

**Additional file 2: Table of characteristics of matched pairs.**

| **Diagnosis** | **Procedure** | **Sex** | **ASA** | **Year Interval** |
| --- | --- | --- | --- | --- |
| **ME/CFS** | **Ophthalmic surgery (phacoemulsification, IOL implantation, ppV)** | **F** | **II** | **2015-2020** |
| Control | Ophthalmic surgery (phacoemulsification, IOL implantation, ppV) | F | II | 2015-2020 |
|  |  |  |  |  |
| **ME/CFS** | **Pansinusotomy** | **F** | **II** | **2015-2020** |
| Control | Pansinusotomy | F | II | 2015-2020 |
|  |  |  |  |  |
| **ME/CFS** | **Dental extraction** | **F** | **III** | **2015-2020** |
| Control | Mandibular cystectomy | F | II | 2015-2020 |
|  |  |  |  |  |
| **ME/CFS** | **Hepatic resection** | **F** | **III** | **2015-2020** |
| Control | Hepatic resection | F | II | 2015-2020 |
|  |  |  |  |  |
| **ME/CFS** | **Hemithyroidectomy** | **F** | **II** | **2020-2024** |
| Control | Hemithyroidectomy | F | II | 2020-2024 |
|  |  |  |  |  |
| **ME/CFS** | **Total hip arthroplasty** | **F** | **II** | **2020-2024** |
| Control | Total hip arthroplasty | F | II | 2025-2026 |
|  |  |  |  |  |
| **ME/CFS** | **Tympanoplasty** | **M** | **II** | **2020-2024** |
| Control | Tympanoplasty | M | I | 2025-2026 |
|  |  |  |  |  |
| **ME/CFS** | **Pterygomandibular abscess drainage** | **M** | **II** | **2020-2024** |
| Control | Pterygomandibular abscess drainage | M | II | 2025-2026 |
|  |  |  |  |  |
| **ME/CFS** | **Ventriculoperitoneal shunt placement** | **F** | **III** | **2020-2024** |
| Control | Ventriculoperitoneal shunt placement | F | III | 2025-2026 |
|  |  |  |  |  |
| **ME/CFS** | **MRI brain under general anesthesia** | **F** | **III** | **2020-2024** |
| Control | MRI brain under general anesthesia | F | II | 2025-2026 |
|  |  |  |  |  |
| **ME/CFS** | **Pansinusotomy** | **M** | **II** | **2020-2024** |
| Control | Pansinusotomy | M | III | 2025-2026 |
|  |  |  |  |  |
| **ME/CFS** | **VATS** | **F** | **III** | **2020-2024** |
| Control | VATS | F | III | 2025-2026 |
|  |  |  |  |  |
| **ME/CFS** | **Lower limb soft tissue debridement** | **F** | **III** | **2025-2026** |
| Control | Lower limb soft tissue debridement | F | III | 2025-2026 |
|  |  |  |  |  |
| **ME/CFS** | **Laparoscopic cholecystectomy** | **F** | **II** | **2025-2026** |
| Control | Laparoscopic cholecystectomy | F | II | 2025-2026 |
|  |  |  |  |  |
| **ME/CFS** | **Ophthalmic surgery (ppV, membrane peeling)** | **M** | **III** | **2025-2026** |
| Control | Ophthalmic surgery (phacoemulsification, IOL implantation) | M | III | 2025-2026 |

Baseline and procedural characteristics of all 15 matched pairs. Each pair consists of one patient with myalgic encephalomyelitis/chronic fatigue syndrome (ME/CFS, bold) and one matched control. Pairs are displayed by 5-year intervals of the ME/CFS procedure. Matching was performed on sex and procedure type. During control selection, age, BMI, and year of procedure were additionally considered to avoid large discrepancies where possible; ASA: American Society of Anesthesiologists physical status classification; BMI: body mass index; IOL: intraocular lens implantation; MRI: magnetic resonance imaging; VATS: video-assisted thoracoscopic surgery; ppV: pars plana vitrectomy.
