# Supplementary material for: Perioperative outcomes in patients with myalgic encephalomyelitis/chronic fatigue syndrome undergoing general anesthesia: a retrospective matched-pair study
Source: BMC Anesthesiol. 2026 Jul 16;26:426. doi: 10.1186/s12871-026-04102-5 (PMC13374260; doi:10.1186/s12871-026-04102-5)
Supplement: Supplementary file 3 — Additional file 3. Preoperative medication classes. Summary of regular preoperative medication classes in ME/CFS patients and matched controls. [file 12871_2026_4102_MOESM3_ESM.docx]

# **Additional file 3: Table of preoperative medication classes in matched pairs**

| **Variable** | **ME/CFS (n=15)** | **Control (n=15)** | **p-value** |
| --- | --- | --- | --- |
| Opioids, n (%) | 4/13 (30.8%) | 0/15 (0%) | 0.125 |
| NSAIDs, n (%) | 0/13 (0%) | 1/15 (6.7%) | 1.000 |
| Metamizole, n (%) | 3/13 (23.1%) | 2/15 (13.3%) | 1.000 |
| Paracetamol, n (%) | 2/13 (15.4%) | 0/15 (0%) | 0.500 |
| SSRI/SNRI, n (%) | 5/13 (38.5%) | 1/15 (6.7%) | 0.125 |
| Other antidepressants (mirtazapine, trazodone, opipramol), n (%) | 3/13 (23.1%) | 1/15 (6.7%) | 0.500 |
| Tricyclic antidepressants, n (%) | 0/13 (0%) | 0/15 (0%) | — |
| Anticonvulsants, n (%) | 0/13 (0%) | 0/15 (0%) | — |
| Cannabis, n (%) | 3/13 (23.1%) | 0/15 (0%) | 0.250 |
| Triptans, n (%) | 0/13 (0%) | 1/15 (6.7%) | — |
| Hypnotics/sedatives, n (%) | 0/13 (0%) | 1/15 (6.7%) | 1.000 |

Binary variables: n/N (%), McNemar's test (exact via binomial test if <10 discordant pairs); — = not tested (no discordant pairs). Preoperative medication data were not documented for 2 patients with ME/CFS (denominators reduced accordingly). Categories are not mutually exclusive; patients on combination regimens are counted in each applicable class. NSAID: non-steroidal anti-inflammatory drug; SSRI: selective serotonin reuptake inhibitor; SNRI: serotonin-noradrenaline reuptake inhibitor.
